# Prognostic value of platelet levels in patients with aneurysmal Subarachnoid Hemorrhage

**DOI:** 10.1038/s41598-024-67322-0

**Published:** 2024-07-20

**Authors:** Igor Fischer, Ronahi Lala, Daniel M. Donaldson, Simon Schieferdecker, Björn B. Hofmann, Jan Frederick Cornelius, Sajjad Muhammad

**Affiliations:** 1 Department of Neurosurgery, Medical Faculty, University Hospital Düsseldorf, Heinrich-Heine-University, Dusseldorf, Germany; 2 Department of Neurosurgery, University of Helsinki and Helsinki University Hospital, Helsinki, Finland; 3 Department of Neurosurgery, King Edward Medical University, Lahore, Pakistan; 4 Department of Anaesthesiology, Medical Faculty, Heinrich-Heine-University Düsseldorf, Dusseldorf, Germany

**Keywords:** SAH, Neuroinflammation, CBC, Predictive model, Prognostic markers, Neurovascular disorders

## Abstract

Pathophysiological processes following aneurysmal subarachnoid hemorrhage (aSAH) include upregulated underlying systemic inflammation, which is reflected by changes in different peripheral blood cells and their sub-populations. As inflammation is a crucial process that contributes to post-aSAH complications and clincal outcome, blood cell numbers and ratios in systemic circulation may predict the outcome and provide rapid and easy to quantify point of care biomarkers for these critically ill patients. To identify blood-derived cellular inflammatory parameters which allow a precise prediction of patient outcome after aSAH. In this single-center retrospective study, 19 whole blood-derived cellular inflammatory markers and clinical and demographic parameters for 101 aSAH patients were recorded within 24 h after aSAH. Clinical outcome was quantified with modified Rankin scale (mRS) on discharge. Proportional odds logistic regression (POLR) was used to model the patient outcome as the function of clinical parameters and inflammatory markers. The results were validated on a separate hold-out dataset (220 patients). The on-admission platelet count, mean platelet volume (MPV) and mean platelet volume to platelet ratio (MPR) were found to be significant and predictive of patient outcome on discharge. Mean platelet volume (MPV) and mean platelet volume to platelet ratio (MPR) predicted clinical outcome and may serve as easy to quantify point of care biomarker. The findings are potentially relevant for the management of aSAH.

## Introduction

Subarachnoid hemorrhage (SAH) is a debilitating and fatal disease. In about 75–85% of the cases SAH is caused by the rupture of an intracranial aneurysm^1,2^. The worldwide incidence estimate lies around 9 per 100,000 person-years, with high regional variability^3,4^. The mortality is high, reportedly around 50%^5^ or even higher. Many survivors suffer from permanent disability, with about 50% dependency rate^6^.

The long-term prognosis and recovery from SAH depend on various factors, including the extent and the localization of the hemorrhage, comorbidities and underlying conditions, and the timeliness of medical intervention. Rehabilitation and supportive care, including physical and cognitive therapy, are often necessary to regain function and manage lasting effects.

Aneurysmal SAH (aSAH) is known to induce a peripheral immune response, leading to the accumulation of immune cells in the brain parenchyma^7,8^. It has been shown that the resulting inflammation correlates with poor clinical status at admission^9^. Successful management of post-aSAH inflammation may therefore offer therapeutic benefits^10–12^.

Despite the large body of literature, the pathophysiological processes surrounding inflammation are complex, convoluted and still not completely understood. A systematic search on PubMed (https://pubmed.ncbi.nlm.nih.gov) found over 1200 articles related to (a)SAH and serum biomarkers, with varying results. To shed more light, we investigated a number of inflammation-specific markers in the serum of patients suffering from aSAH and correlated them to the clinical outcome with overarching aim to find easy to quantify point of care diagnostic biomarkers for disease progression including detection of post SAH complications.

Other studies, like^13^, have investigated numerous serum biomarkers and their association with the patient outcome. Large amounts of clinical and laboratory data collected for each patient make it enticing to look into possible correlations, with the danger of also discovering spurious ones. Such models may appear to be in good agreement with the data used in the modelling phase but perform poorly in practice. While this danger cannot be completely avoided, it can be mitigated. An established procedure is to use separate datasets in different modelling phases^14^.

Nevertheless, easy to quantify diagnostic blood biomarkers could be highly relevant to guide treatment decisions during post SAH care. For our study we have chosen markers which are routinely collected in medical practice and easy to analyse, to allow for quick clinical decisions.

## Methods

### Study type and population

This is a single-center retrospective study. The cohort consisted of aSAH patients admitted to our quaternary university hospital between June 2019 and September 2022 and of patients admitted between January 2014 and December 2016. The former, exploratory dataset was used to identify potential predictors and the latter to confirm the findings. Clinical parameters and full blood count were determined within 24 h after the onset of the aSAH. The patients with age ≤ 18 years, ischemic stroke, traumatic brain injury, onset of symptoms beyond 24 h, SAH due to arteriovenous malformations or vasculitis, pregnancy, signs of imminent death, or those who did not provide consent were excluded from the study. Altogether, the exploratory dataset comprised 101 patients and the confirmatory one 220. The latter was compiled only after the completion of the exploratory phase. The demographic variables, clinical parameters, and the corresponding descriptive statistics are listed in Table 1. Seventy-one patients whose data were used for exploratory analysis required extraventricular drainage (EVD), 20 vasospasms, and 47 pneumonia. Of the 17 patients who died in hospital, in 11 cases (65%), in accordance with the clearly stated patient’s will, life-sustaining treatment was withdrawn and the therapy changed to palliative. No significant differences were found between the exploratory and the confirmatory dataset (Table 2).

**Table 1:** Patient demographics, total and by outcome group, for the exploratory and the confirmatory set combined.

Variable	Total	mRS ≤ 2	3 ≤﻿ mRS ≤﻿ 5	mRS = 6	p
Mean (sd)					p
Age	57 (12)	52 (11)	58 (11)	63 (13)	< .0001
Levels					
Sex	n (%)	n (%)	n (%)	n (%)	p
Female	211 (66%)	70 (63%)	106 (69%)	35 (61%)	0.4
Male	110 (34%)	41 (37%)	47 (31%)	22 (39%)	
WFNS	n (%)	n (%)	n (%)	n (%)	p
1	102 (32%)	68 (61%)	32 (21%)	2 (4%)	< .0001
2	50 (16%)	25 (23%)	17 (11%)	8 (14%)	
3	11 (3%)	3 (3%)	6 (4%)	2 (4%)	
4	62 (19%)	12 (11%)	40 (26%)	10 (18%)	
5	96 (30%)	3 (3%)	58 (38%)	35 (61%)	
Modified Fisher	n (%)	n (%)	n (%)	n (%)	p
0	6 (2%)	3 (3%)	3 (2%)	0 (0%)	< .0001
1	19 (6%)	15 (14%)	4 (3%)	0 (0%)	
2	33 (10%)	6 (5%)	20 (13%)	7 (12%)	
3	68 (21%)	35 (32%)	25 (16%)	8 (14%)	
4	194 (61%)	52 (47%)	100 (66%)	42 (74%)	
Vasospasm	n (%)	n (%)	n (%)	n (%)	p
No	245 (77%)	91 (82%)	106 (70%)	48 (84%)	0.03
Yes	74 (23%)	20 (18%)	45 (30%)	9 (16%)	
Pneumonia	n (%)	n (%)	n (%)	n (%)	p
No	191 (60%)	101 (91%)	54 (36%)	36 (63%)	0.008
Yes	129 (40%)	10 (9%)	98 (64%)	21 (37%)	
EVD	n (%)	n (%)	n (%)	n (%)	p
No	94 (33%)	65 (66%)	19 (14%)	10 (20%)	< .0001
Yes	188 (67%)	33 (34%)	116 (86%)	39 (80%)	
CNS infection	n (%)	n (%)	n (%)	n (%)	p
No	280 (88%)	102 (92%)	124 (82%)	54 (95%)	0.008
Yes	40 (12%)	9 (8%)	28 (18%)	3 (5%)	
Urogenital infection	n (%)	n (%)	n (%)	n (%)	p
No	307 (96%)	109 (98%)	142 (93%)	56 (98%)	0.1
Yes	13 (4%)	2 (2%)	10 (7%)	1 (2%)	
Epilepsy	n (%)	n (%)	n (%)	n (%)	p
No	257 (80%)	107 (96%)	107 (70%)	43 (75%)	< .0001
Yes	63 (20%)	4 (4%)	45 (30%)	14 (25%)	
Myocardial infarction	n (%)	n (%)	n (%)	n (%)	p
No	314 (98%)	111 (100%)	148 (97%)	55 (96%)	0.2
Yes	6 (2%)	0 (0%)	4 (3%)	2 (4%)	
VPS	n (%)	n (%)	n (%)	n (%)	p
No	212 (66%)	96 (86%)	63 (41%)	53 (93%)	< .0001
Yes	108 (34%)	15 (14%)	89 (59%)	4 (7%)	
Early ischemia	n (%)	n (%)	n (%)	n (%)	p
No	238 (74%)	111 (100%)	106 (70%)	21 (37%)	< .0001
Yes	82 (26%)	0 (0%)	46 (30%)	36 (63%)	
CPR	n (%)	n (%)	n (%)	n (%)	p
No	305 (96%)	111 (100%)	147 (97%)	47 (82%)	< .0001
Yes	14 (4%)	0 (0%)	4 (3%)	10 (18%)	
ICH	n (%)	n (%)	n (%)	n (%)	p
≤﻿ 10 ml	210 (65%)	101 (91%)	82 (54%)	27 (47%)	< .0001
> 10 ml	111 (35%)	10 (9%)	71 (46%)	30 (53%)	
IVH	n (%)	n (%)	n (%)	n (%)	p
No	94 (29%)	53 (48%)	33 (22%)	8 (14%)	< .0001
Yes	227 (71%)	58 (52%)	120 (78%)	49 (86%)	
DCI (*)	n (%)	n (%)	n (%)	n (%)	p
No	79 (79%)	26 (84%)	37 (71%)	16 (94%)	0.09
Yes	21 (21%)	5 (16%)	15 (29%)	1 (6%)	

The entries marked with an asterisk (*) refer only to the exploratory set.

**Table 2:** Patient demographics, total and by dataset used in analysis.

Variable	Total	confirmatory	exploratory	p
Mean (sd)				
Age	57 (12)	57 (12)	57 (12)	0.7
Levels				
Sex	n (%)	n (%)	n (%)	p
Female	211 (66%)	143 (65%)	68 (67%)	0.8
Male	110 (34%)	77 (35%)	33 (33%)	
WFNS	n (%)	n (%)	n (%)	p
1	102 (32%)	72 (33%)	30 (30%)	0.8
2	50 (16%)	33 (15%)	17 (17%)	
3	11 (3%)	9 (4%)	2 (2%)	
4	62 (19%)	42 (19%)	20 (20%)	
5	96 (30%)	64 (29%)	32 (32%)	
Modified Fisher	n (%)	n (%)	n (%)	p
0	6 (2%)	4 (2%)	2 (2%)	0.9
1	19 (6%)	12 (5%)	7 (7%)	
2	33 (10%)	24 (11%)	9 (9%)	
3	68 (21%)	45 (20%)	23 (23%)	
4	194 (61%)	135 (61%)	59 (59%)	
mRS	n (%)	n (%)	n (%)	p
0–2	111 (35%)	80 (36%)	31 (31%)	0.5
3–5	153 (48%)	100 (45%)	53 (52%)	
6	57 (18%)	40 (18%)	17 (17%)	
Vasospasm	n (%)	n (%)	n (%)	p
No	245 (77%)	166 (75%)	79 (80%)	0.5
Yes	74 (23%)	54 (25%)	20 (20%)	
Pneumonia	n (%)	n (%)	n (%)	p
No	191 (60%)	138 (63%)	53 (53%)	0.1
Yes	129 (40%)	82 (37%)	47 (47%)	
EVD	n (%)	n (%)	n (%)	p
No	94 (33%)	64 (35%)	30 (30%)	0.4
Yes	188 (67%)	117 (65%)	71 (70%)	
Aneurysm location	n (%)	n (%)	n (%)	p
ACI	19 (6%)	12 (5%)	7 (7%)	0.2
ACOM	133 (41%)	83 (38%)	50 (50%)	
Anterior choroidal artery	4 (1%)	2 (1%)	2 (2%)	
Basilar artery	26 (8%)	16 (7%)	10 (10%)	
MCA	66 (21%)	50 (23%)	16 (16%)	
PCA	2 (1%)	2 (1%)	0 (0%)	
PCOM	31 (10%)	27 (12%)	4 (4%)	
PICA	14 (4%)	10 (5%)	4 (4%)	
Pericallosal artery	12 (4%)	8 (4%)	4 (4%)	
Vertebral artery	8 (2%)	7 (3%)	1 (1%)	
Other	6 (2%)	3 (1%)	3 (3%)	
Life-support withdrawn	n (%)	n (%)	n (%)	p
No	11 (20%)	5 (13%)	6 (35%)	0.1
Yes	44 (80%)	33 (87%)	11 (65%)	

p > .05 indicates no significant differences between the subsets.

### Variable definition and data collection

**Figure 1:**
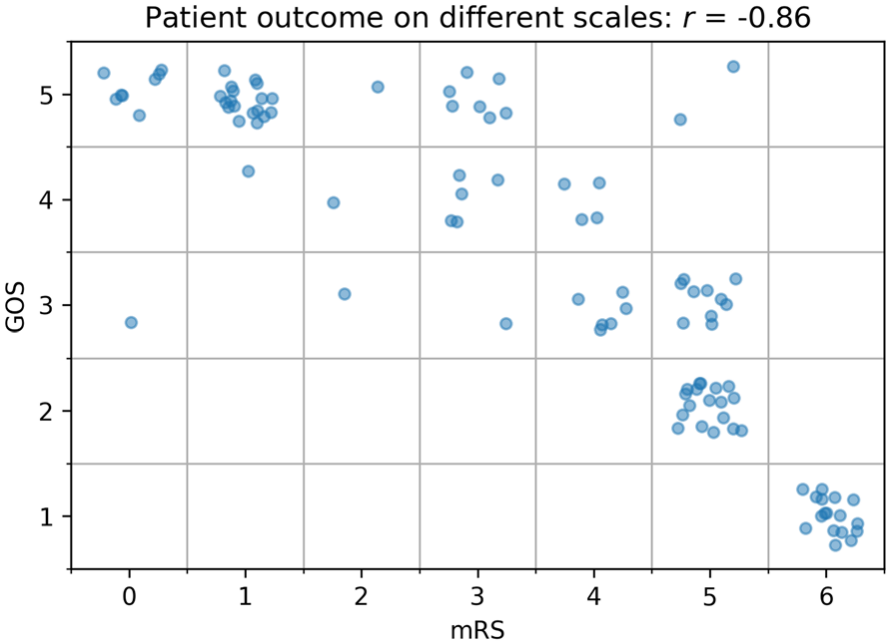
Relationship measures of outcome, modified Rankin scale (mRS) and Glasgow outcome scale (GOS), for the exploratory dataset. Each dot represents a patient.

Patients’ demographic and clinical data were recorded at admission. For the exploratory analysis, eighteen CBC-derived inflammation blood markers (Table 3) were selected from the blood analysis performed at the central hospital laboratory. The markers which were found to be significant were used in subsequent confirmatory analysis. SAH was determined by computerized tomography (CT) scan and evidence of an aneurysm was subsequently confirmed by CT angiography or digital subtraction angiography. Patient outcome at discharge was quantified using the Glasgow outcome scale (GOS) and modified Rankin scale (mRS) at discharge (Fig. 1).

**Table 3:** Marker characteristics for the patients from the exploratory set, total and by outcome group.

Variable	Total	mRS ≤﻿ 2	3 ≤﻿ mRS ≤﻿ 5	mRS = 6
Mean (sd)				
WBC	14.2 (5.0)	13.9 (5.0)	13.7 (3.9)	16.7 (7.2)
MPV	10.38 (0.87)	10.23 (0.84)	10.37 (0.87)	10.73 (0.89)
platelets	231 (63)	240 (69)	232 (55)	209 (72)
NLR	12.8 (8.2)	10.5 (8.0)	13.5 (7.8)	15.1 (8.9)
dNLR	6.3 (4.0)	5.6 (3.2)	6.0 (3.6)	8.8 (5.6)
PLR	240 (130)	210 (120)	260 (140)	230 (120)
MPR	0.049 (0.018)	0.046 (0.014)	0.048 (0.015)	0.059 (0.027)
NLPR	0.061 (0.046)	0.045 (0.037)	0.061 (0.038)	0.085 (0.070)
MLR	0.75 (0.54)	0.63 (0.38)	0.81 (0.65)	0.78 (0.36)
SIRI	9.4 (7.6)	7.9 (6.9)	9.9 (8.5)	10.5 (6.2)
SII	2900 (1900)	2600 (2000)	3100 (1900)	3100 (1900)
AISI	2200 (1900)	2000 (1900)	2300 (2000)	2200 (1600)
Lymphocytes (%)	9.6 (6.4)	11.8 (6.9)	9.2 (6.3)	6.4 (3.7)
Monocytes (%)	5.8 (3.0)	6.0 (2.3)	5.9 (3.6)	5.1 (1.9)
Neutrophils (%)	87 (34)	81 (9)	90 (47)	87 (5)
Eosinophils (%)	0.40 (0.87)	0.62 (1.24)	0.37 (0.72)	0.11 (0.14)
Basophils (%)	0.38 (0.44)	0.48 (0.67)	0.32 (0.17)	0.38 (0.48)
Bilirubin	0.46 (0.26)	0.47 (0.26)	0.43 (0.23)	0.53 (0.33)

### Data analysis

The outcome was quantified by modified Rankin scale (mRS) at discharge and summarized in three ordered categories: favorable (0 ≤﻿ mRS ≤﻿ 2), poor (3 ≤﻿ mRS ≤﻿ 5), and death (mRS = 6). The said boundary between favorable and poor outcome is the most common in the literature^15^. Age, sex, the presence of extraventricular drainage (EVD), DCI, pneumonia, or vasospasms, the modified Fisher score, the WFNS score, and all inflammation markers listed in Table 3 were treated as potential predictor variables in proportional odds logistic models.

The WFNS score, which is easily and routinely determined, is known to be highly correlated with or even the most predictive parameter for the patient outcome^16–19^. In order to enforce our models to outperform it, we decided to use it as a fixed predictor in every model. Next, in the exploratory phase, a series of models with one additional predictor was fitted. Significant predictors (p < 0.05)—i.e. those which improved the simple, WFNS-only model—were recorded. Colinearity between predictors was tested using linear regression. The findings from the exploratory phase were validated on the confirmatory dataset. The dependence between a single predictor variable and the trichotomized outcome was analyzed using one-way ANOVA. If ANOVA indicated significant differences between outcome levels, post-hoc t-test was used to identify them. For multiple predictor variables, the dependency was modeled by proportional odds logistic regression (POLR). Multiple regression was performed using all significant predictors found by univariate analysis.

All computations were performed in Python (www.python.org), using NumPy^20^, SciPy^21^, and Statsmodels^22^ libraries.

### Ethical approval

The study was performed according to the guidelines of the Helsinki declaration and was approved by the local ethics committee of the medical faculty of the Heinrich-Heine-University Düsseldorf, Germany (reference number: 2019-787-bio). The informed consent was obtained by the treating neurosurgeon. The manuscript was prepared according to the STROBE guidelines.

## Results

In the exploratory phase, WFNS and age were shown to be highly significant predictors for the outcome (p < 0.0001 for both). We also identified platelets, MPV, and MPR as significant predictors (p < 0.05), even when taken as a second independent variable next to the WFNS score. The three markers were strongly linearly related (p < 0.001), which is unsurprising, given that all three are related to platelets. In particular, the inverse of the MPR was nearly perfectly colinear with platelets (R^2^ = 0.941). In the confirmatory phase, using a separate dataset, all three markers proved to be significant predictors (POLR, p = 0.009, p = 0.013 and p = 0.011 for platelets, MPV and MPR, respectively). No significant difference in the distribution of the markers was found between the two sets (Table 4). All three markers were also colinear with the WFNS score (p = 0.02, p = 0.04, and p = 0.005) and, in addition, platelets count and MPR were colinear with age (p < 0.0001 and p = 0.0001, respectively). No collinearity between MPV and age was observed (p = 0.4).

**Table 4:** Marker characteristics, total and by dataset used in analysis.

Variable	Total	Confirmatory	Exploratory	p
Mean (sd)				
platelets	237 (68)	240 (70)	231 (63)	0.3
MPV	10.43 (0.91)	10.46 (0.93)	10.38 (0.87)	0.5
MPR	0.049 (0.023)	0.048 (0.025)	0.049 (0.018)	0.8

p > .05 indicates no significant differences between the subsets.

**Figure 2:**
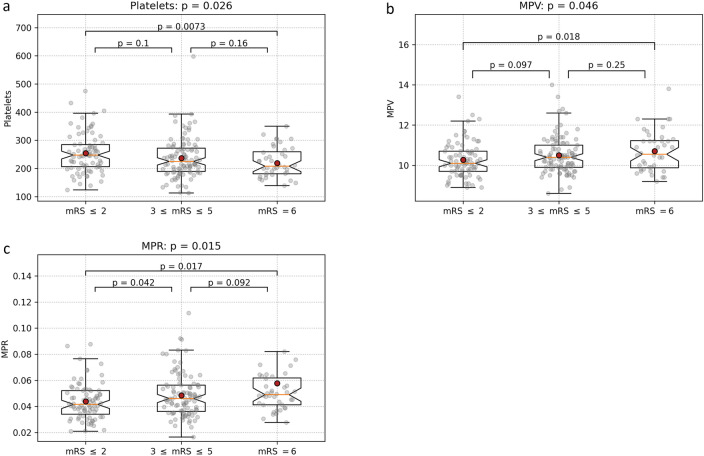
Association between platelet-related markers and the patient outcome (trichotomized mRS score): (**a**) platelet count; (**b**) MPV; (**c**) MPR. Only MPR showed a significant difference between patients with a favorable outcome and both other groups. The difference between poor outcome and death was not significant, but a trend (p < 0.1).

However, ANOVA and the post-hoc t-tests showed that these three markers didn’t significantly differ over all outcome levels. Platelet levels differed significantly only between patients with favorable outcome and the deceased (p = 0.007), but not between the patients with poor outcome and the other two groups. A similar result was observed for MPV (p = 0.018 for differences between favorable outcome and death), but there was also a trend (p < 0.1) between the groups with favorable and poor outcome. MPR showed the best differentiation capability, with significant differences between favorable on one side and both poor outcome and death on the other (p = 0.042 and p = 0.017, respectively). There was also a statistical trend (p < 0.1) between MPR values for patients with poor outcome and the deceased (Fig. 2a–c).

**Figure 3:**
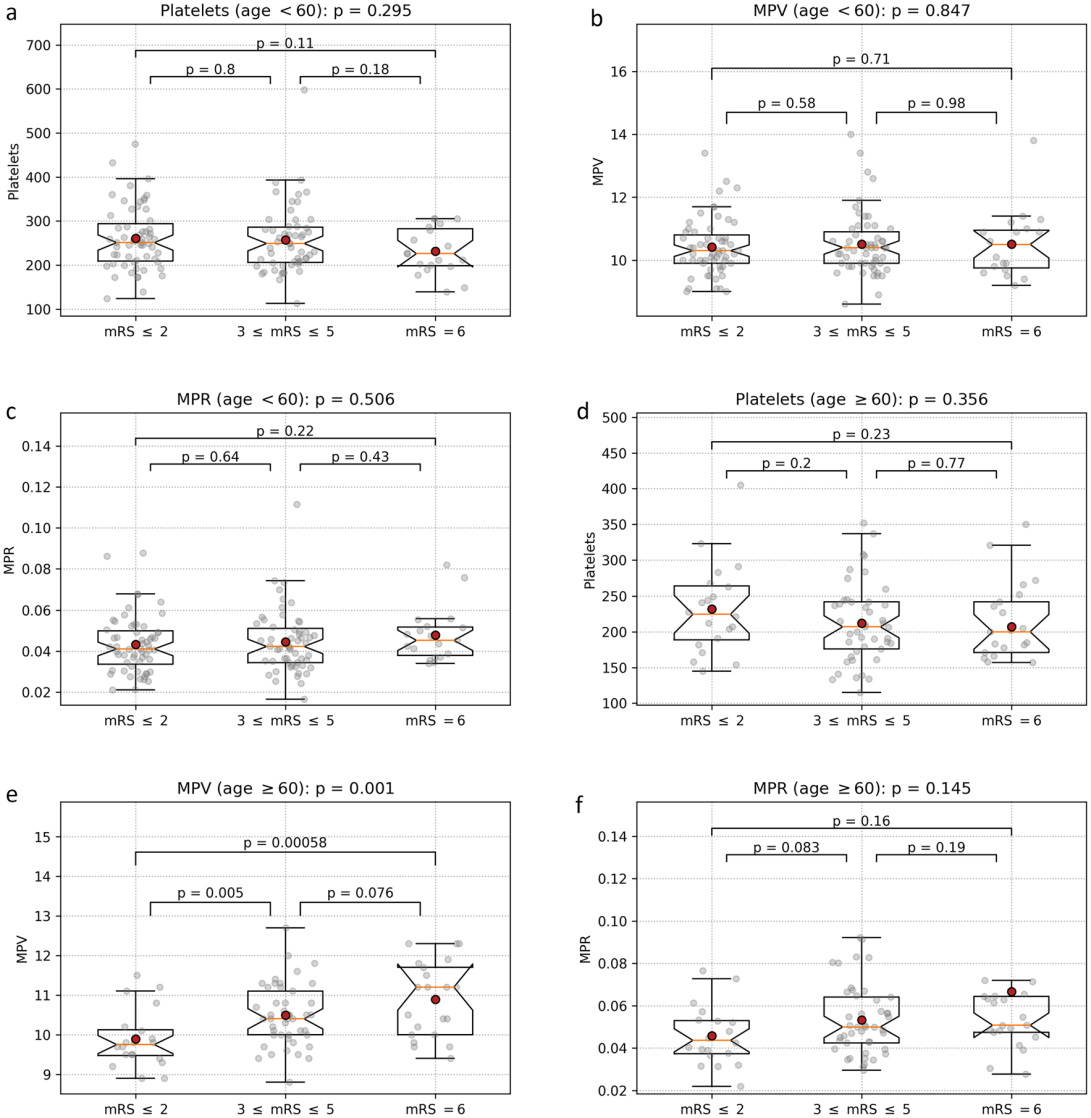
Subgroup analysis by age for the platelet-related markers: (**a**) platelet count; (**b**) MPV; (**c**) MPR, all three for patients under 60. Panels (**d**–**f**) show the same for patients aged 60 and more. A significant difference can only be observed for MPV in the latter group, but the directions of change for values between outcome groups remain the same.

As age is an important factor for overall health, whose impact rises strongly as it advances, we analyzed the association between the three predictors and the outcome separately for two groups: patients under 60 (133 patients) and patients aged 60 and over (86 patients) (Fig. 3a–f). Although the trends remain the same—decreasing platelet counts and increasing MPV and MPR for worse outcomes—the differences are significant only for MPV for the elderly patients. In part, this can be explained by the reduction in set sizes, as small effects are statistically harder to detect on smaller sets. MPV, however, turns to be a much better predictor in the elderly group than for all patients taken together, with highly significant differences (p < 0.01) between patients with favorable outcome and the other two groups, and a trend (p = 0.076) between unfavorable outcome and the deceased.

**Figure 4:**
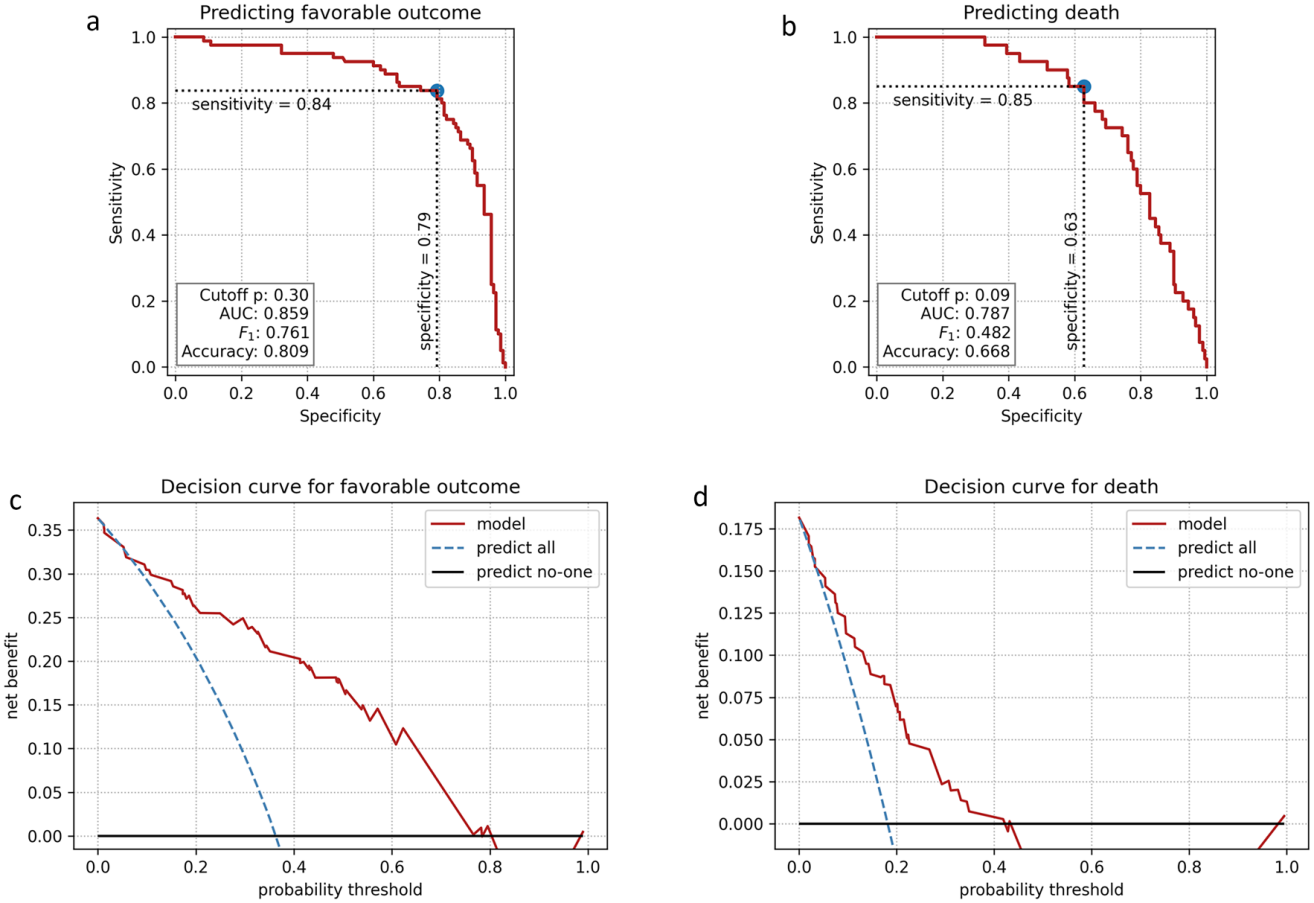
Receiver operating characteristics (ROC), (**a**) for predicting favorable outcome and (**b**) for predicting death. Panels (**c**) and (**d**) show the respective decision curves.

In the multiple regression model, using the WFNS score, the age, and all three markers as the predictors, platelets and MPR turned out not to be significant, and a trend (p = 0.07) was observed for MPV. This loss of significance was expected, due to the above collinearities. Figure 4a–d show the ROC curves and the decision curves for predicting favorable outcome and death using the multiple regression model.

## Discussion

We investigated several known blood markers involved in inflammation processes, together with clinical parameters, for their predictivity of patient outcome after aSAH. As the number of markers was substantial in relation to the data size, special attention was paid to ensure that the results are reliable and not resulting from random noise in the data. We have therefore reason to believe that our findings are generalizable beyond data used in this study.

Patient’s initial status, reflected in the WFNS score, is—unsurprisingly—higly predictive of the outcome, confirming the ample evidence reported in the literature. Patients with a low initial WFNS score tend to recover better, while patients with a higher WFNS score or poor clinical status at admission are likely to have a worse outcome. At the same time, the WFNS score is plagued by subjectivity and inaccuracies in early assessment and can be supplemented by laboratory results. The role of platelet count and related indicators, MPV and MPR, needs to be explained within the body of the available knowledge. The current literature on platelets and prediction of post SAH complications is controversial.

While some studies indicate that MPR might serve as a predictor of DCI or overall outcome after aSAH^23,24^, others haven’t found such an association^25^. A recent study found no association between platelet count and DCI after aSAH^26^. We applied different ratios of blood cells including mean platelet volume (MPV) and mean platelet volume to platelet ratio (MPR) to evaluate the predictive value of systemic platelet numbers and consequently platelet mediated inflammation. The association between MPR and patient outcome, found in our study, is significant to differentiate and classify different outcome groups (good vs poor outcome) based on modified Rankin scale. Our analysed ratios, however, were not sufficient to differentiate between poor outcome group and the group of patient with in-hospital death. This might be due to high spread of MPR values in the group with poor outcome, causing a significant overlap with the values in the smaller group of deceased patients (Fig. 2c). By its definition, MPR is bound to vary more when the platelet counts are low, and this tends to be the case for patients with poor outcome or death (Fig. 2a), although only the difference between favorable outcome and death was significant. Other studies^27,28^ found no significant association between the platelet count and the outcome, but the raw numbers point in the same direction. The latter study found a significant negative correlation between platelets and the SAH volume, suggesting a possible link.

Altogether, our findings suggests that MPR might play a secondary role as the outcome predictor. Nevertheless, combining MPV and MPR can be a valuable tool to rapidly recognize the patients with poor outcome after SAH and hence, may help in treatment decision making during intensive care.

### Limitations

The study was retrospective by design, based on a single-center cohort. The primary outcome (mRS) was determined retrospectively based on the medical records. We only investigated mRS at discharge, which is indicative of, but not the same as the long-term outcome, e.g. six months after discharge. Although we used two different datasets for exploration and confirmation, further studies on external and ideally larger cohorts are needed to corroborate the findings. Investigating the distribution of predictor values for different outcome categories showed that log-odds for “death” violate the linearity assumption, thus weakening the predictions. Nevertheless, the overall direction points in the same way as under the linearity assumption. Also, the study was phenomenological and further basic research into the exact mechanisms of inflammation and its consequences is warranted.

## Conclusion

Three easy-to-determine inflammation markers, platelet count, MPV, and MPR, are related to patient outcome after suffering aSAH. Together with the WFNS score, they can be used to indicate whether a patient is at a higher-than-expected risk of poor outcome and adjust the treatment as needed.

## Data Availability

The datasets analyzed during the current study are available from the corresponding author on reasonable request.

## Abbreviations

AISI: Aggregate index of systemic inflammation
aSAH: Aneurysmal subarachnoid hemorrhage
AUC: Area under the curve
CBC: Complete blood count
CT: Computerized tomography
DCI: Delayed cerebral ischemia
dNLR: Derived neutrophil-to-lymphocyte ratio
EVD: Extra-ventricular drainage
GOS: Glasgow outcome scale
MLR: Monocyte-to-lymphocyte ratio
MPV: Mean platelet volume
MPR: Mean platelet volume to platelet ratio
mRS: Modified Rankin scale
NLR: Neutrophil-to-lymphocyte ratio
NLPR: Neutrophil-to-lymphocyte times platelet ratio
PLR: Platelet-to-lymphocyte ratio
ROC: Receiver operating characteristics
SII: Systemic inflammation index
SIRI: Systemic inflammation response index
WBC: White blood cell count
WFNS: World federation of neurosurgeons

## References

[1] Priebe, H. Aneurysmal subarachnoid haemorrhage and the anaesthetist. *Br. J. Anaesth.* **99**(1), 102–118 (2007). 17525049 10.1093/bja/aem119

[2] van Gijn, J. & Rinkel, G. Subarachnoid haemorrhage: Diagnosis, causes and management. *Brain* **124**(Pt 2), 249–278 (2001). 11157554 10.1093/brain/124.2.249

[3] Linn, F., Rinkel, G., Algra, A. & van Gijn, J. Incidence of subarachnoid hemorrhage: Role of region, year, and rate of computed tomography: A meta-analysis. *Stroke* **27**(4), 625–629 (1996). 8614919 10.1161/01.STR.27.4.625

[4] de Rooij, N., Linn, F., van der Plas, J., Algra, A. & Rinkel, G. Incidence of subarachnoid haemorrhage: A systematic review with emphasis on region, age, gender and time trends. *J. Neurol. Neurosurg. Psychiatry* **78**(12), 1365–1372 (2007). 17470467 10.1136/jnnp.2007.117655 PMC2095631

[5] Long, B., Koyfman, A. & Runyon, M. Subarachnoid hemorrhage: Updates in diagnosis and management. *Emerg. Med. Clin. N. Am.* **35**(4), 803–824 (2017). 10.1016/j.emc.2017.07.001 28987430

[6] le Roux, A. & Wallace, M. Outcome and cost of aneurysmal subarachnoid hemorrhage. *Neurosurg. Clin. N. Am.* **21**(2), 235–246 (2010). 20380966 10.1016/j.nec.2009.10.014

[7] Moraes, L. *et al.* Immune cells subpopulations in cerebrospinal fluid and peripheral blood of patients with Aneurysmal Subarachnoid Hemorrhage. *Springerplus* **4**, 195 (2015). 25977890 10.1186/s40064-015-0970-2 PMC4414856

[8] Chaudhry, S. R. *et al.* Differential polarization and activation dynamics of systemic T helper cell subsets after aneurysmal subarachnoid hemorrhage (SAH) and during post-SAH complications. *Sci. Rep.* **11**, 14226 (2021). 34244562 10.1038/s41598-021-92873-x PMC8270974

[9] Savarraj, J. P. J. *et al.* Systematic model of peripheral inflammation after subarachnoid hemorrhage. *Neurology* **88**(16), 1535–1545 (2017). 28314864 10.1212/WNL.0000000000003842 PMC5395070

[10] Ahn, S.-H. *et al.* Inflammation in delayed ischemia and functional outcomes after subarachnoid hemorrhage. *J. Neuroinflamm.* **16**, 213 (2019). 10.1186/s12974-019-1578-1 PMC6849179 31711504

[11] Muhammad, S. & Hänggi, D. Inflammation and anti-inflammatory targets after aneurysmal subarachnoid hemorrhage. *Int. J. Mol. Sci* **22**(14), 7355 (2021). 34298971 10.3390/ijms22147355 PMC8304004

[12] Muhammad, S. *et al.* Targeting high mobility group box 1 in subarachnoid hemorrhage: A systematic review. *Int. J. Mol. Sci.* **21**(8), 2709 (2020). 32295146 10.3390/ijms21082709 PMC7215307

[13] Nie, Z., Lin, F., Li, R., Chen, X. & Zhao, Y. A pooled analysis of preoperative inflammatory biomarkers to predict 90-day outcomes in patients with an aneurysmal subarachnoid hemorrhage: A single-center retrospective study. *Brain Sci.* **13**(2), 257 (2023). 36831800 10.3390/brainsci13020257 PMC9954360

[14] Johnson, R. A. & Wichern, D. W. *Applied Multivariate Statistical Analysis* 595 (Pearson Prentice Hall, 2007).

[15] Andersen, C. R., Fitzgerald, E., Delaney, A. & Finfer, S. A systematic review of outcome measures employed in aneurysmal subarachnoid hemorrhage (aSAH) clinical research. *Neurocrit. Care* **30**, 534–541 (2018). 10.1007/s12028-018-0566-0 29951958

[16] Zhao, L. *et al.* Postoperative red blood cell distribution width predicts functional outcome in aneurysmal subarachnoid hemorrhage after surgical clipping: A single-center retrospective study. *Front. Neurol.* **13**, 1036433 (2022). 36619907 10.3389/fneur.2022.1036433 PMC9817139

[17] Hammer, A. *et al.* Dynamics of outcome after aneurysmal subarachnoid hemorrhage. *Aging* **12**(8), 7207–7217 (2020). 32312942 10.18632/aging.103069 PMC7202490

[18] Nguyen, T. *et al.* Predictive validity of the prognosis on admission aneurysmal subarachnoid haemorrhage scale for the outcome of patients with aneurysmal subarachnoid haemorrhage. *Sci. Rep.* **13**(1), 6721 (2023). 37185953 10.1038/s41598-023-33798-5 PMC10130082

[19] Hofmann, B. B. *et al.* Clinical outcome prediction of early brain injury in aneurysmal subarachnoid hemorrhage: The SHELTER-Score. *Neurocrit. Care* **40**(2), 438–447 (2024). 38030877 10.1007/s12028-023-01879-y PMC10959788

[20] Harris, C. *et al.* Array programming with NumPy. *Nature* **585**, 357–362 (2020). 32939066 10.1038/s41586-020-2649-2 PMC7759461

[21] Virtanen, P. *et al.* SciPy 1.0: Fundamental Algorithms for scientific computing in python. *Nat. Methods* **17**(3), 261–272 (2020). 32015543 10.1038/s41592-019-0686-2 PMC7056644

[22] Seabold, S. & J. Perktold, J. Econometric and statistical modeling with Python. In *9th Python in Science Conference*, 2010.

[23] Ray, B. *et al.* Trends of platelet volume index predicts delayed cerebral ischemia after subarachnoid hemorrhage. *World Neurosurg.* **111**, e624–e631 (2018). 29292187 10.1016/j.wneu.2017.12.131

[24] Wang, Z., Pei, W., Chen, L., Ning, Y. & Luo, Y. Mean platelet volume/platelet count ratio is associated with poor clinical outcome after aneurysmal subarachnoid hemorrhage. *J. Stroke Cerebrovasc. Dis.* **29**(11), 105208 (2020). 33066948 10.1016/j.jstrokecerebrovasdis.2020.105208

[25] Ray, B. *et al.* Systemic response of coated-platelet and peripheral blood inflammatory cell indices after aneurysmal subarachnoid hemorrhage and long-term clinical outcome. *J. Crit. Care* **52**, 1–9 (2019). 30904732 10.1016/j.jcrc.2019.03.003 PMC8663918

[26] Raatikainen, E. *et al.* Platelet count is not associated with delayed cerebral ischemia after aneurysmal subarachnoid hemorrhage as defined by the 2010 consensus definition. *J. Neurol. Sci.* **436**, 120227 (2022). 35334421 10.1016/j.jns.2022.120227

[27] Yun, S., Yi, H. J., Lee, D. H. & Sung, J. H. Clinical significance of platelet to neutrophil ratio and platelet to lymphocyte ratio in patients with aneurysmal subarachnoid hemorrhage. *J. Clin. Neurosci.* **92**, 49–54 (2021). 34509261 10.1016/j.jocn.2021.07.036

[28] Rzepliński, R., Kostyra, K., Skadorwa, T., Sługocki, M. & Kostkiewicz, B. Acute platelet response to aneurysmal subarachnoid hemorrhage depends on severity and distribution of bleeding: An observational cohort study. *Neurosurg. Rev.* **44**, 2647–2658 (2021). 33241455 10.1007/s10143-020-01444-7

